# Neuroprotective and Cardiometabolic Role of Vitamin E: Alleviating Neuroinflammation and Metabolic Disturbance Induced by AlCl_3_ in Rat Models

**DOI:** 10.3390/biomedicines11092453

**Published:** 2023-09-04

**Authors:** Komal Jabeen, Kanwal Rehman, Muhammad Sajid Hamid Akash, Ahmed Nadeem, Tahir Maqbool Mir

**Affiliations:** 1 Institute of Physiology and Pharmacology, University of Agriculture, Faisalabad 38000, Pakistan; 2 Department of Pharmacy, Niazi Medical and Dental College, Sargodha 40100, Pakistan; 3 Department of Pharmacy, The Women University, Multan 66000, Pakistan; 4 Department of Pharmaceutical Chemistry, Government College University, Faisalabad 38000, Pakistan; 5 Department of Pharmacology and Toxicology, College of Pharmacy, King Saud University, Riyadh 11451, Saudi Arabia; 6 National Center for Natural Products Research, School of Pharmacy, University of Mississippi, University, MS 38677, USA

**Keywords:** neuroinflammation, cardiometabolic disturbance, immunohistochemistry, Masson’s trichrome staining

## Abstract

Cardiovascular diseases (CVDs) and neurodegenerative disorders, such as diabetes mellitus and Alzheimer’s disease, share a common pathophysiological link involving insulin resistance (IR), inflammation, and hypertension. Aluminium chloride (AlCl_3_), a known neurotoxicant, has been associated with neurodegeneration, cognitive impairment, and various organ dysfunctions due to the production of reactive oxygen species (ROS) and oxidative stress. In this study, we aimed to investigate the potential protective effects of metformin and vitamin E against AlCl_3_-induced neuroinflammation and cardiometabolic disturbances in rat models. Rats were divided into five groups: a normal control group, an AlCl_3_-treated diseased group without any treatment, and three groups exposed to AlCl_3_ and subsequently administered with metformin (100 mg/kg/day) alone, vitamin E (150 mg/kg/day) orally alone, or a combination of metformin (100 mg/kg/day) and vitamin E (150 mg/kg/day) for 45 days. We analyzed serum biomarkers and histopathological changes in brain, heart, and pancreatic tissues using H&E and Masson’s trichrome staining and immunohistochemistry (IHC). Electrocardiogram (ECG) patterns were observed for all groups. The AlCl_3_-treated group showed elevated levels of inflammatory biomarkers, MDA, and disturbances in glycemic and lipid profiles, along with reduced insulin levels. However, treatment with the combination of metformin and vitamin E resulted in significantly reduced glucose, cholesterol, LDL, and TG levels, accompanied by increased insulin and HDL levels compared to the individual treatment groups. Histopathological analyses revealed that combination therapy preserved neuronal structures, muscle cell nuclei, and normal morphology in the brain, heart, and pancreatic tissues. IHC demonstrated reduced amyloid plaques and neurofibrillary tangles in the combination-treated group compared to the AlCl_3_-treated group. Moreover, the combination group showed a normal ECG pattern, contrasting the altered pattern observed in the AlCl_3_-treated group. Overall, our findings suggest that metformin and vitamin E, in combination, possess neuroprotective and cardiometabolic effects, alleviating AlCl_3_-induced neuroinflammation and metabolic disturbances.

## 1. Introduction

Neuroinflammation plays a critical role in cardiometabolic disturbances, with chronic brain inflammation leading to immune activation and release of pro-inflammatory cytokines like IL-6. These mediators can impact peripheral organs, causing insulin resistance (IR), dyslipidemia, and affecting cardiovascular function. Understanding this association is essential for developing therapeutic strategies that target neuroinflammation alongside traditional metabolic pathways to manage cardiometabolic diseases and potentially delay neurodegenerative disorders like Alzheimer’s disease (AD). This study’s results on metformin and vitamin E’s protective effects offer valuable insights into potential treatments that modulate both brain and peripheral organ function to promote overall health.

Cardiometabolic disorder is a complex condition characterized by a cluster of metabolic abnormalities and cardiovascular diseases (CVDs). The precise underlying pathways responsible for its development remain unclear; however, IR is believed to be a significant contributor to cardiometabolic disorder. IR poses a substantial risk factor for type 2 diabetes (T2DM), leading to renal sodium ion reabsorption and vasoconstriction and ultimately resulting in hypertension. Common features of cardiometabolic disorder include obesity, hyperinsulinemia, dyslipidemia, and hypertension [1]. On the other hand, AD is a neurodegenerative disorder characterized by the degeneration of cortical and hippocampal neurons, leading to cognitive dysfunction, learning deficits, and memory impairment. AD has been linked to dementia in adults and elders, with global estimates showing that more than 40 million people were affected by dementia in 2016 [2]. The pathogenesis of AD involves the accumulation of intracellular neurofibrillary tangles and extracellular amyloid plaques, resulting in extensive neuronal damage and loss of functional activity in affected individuals. Additionally, AD is associated with various pathological attributes, including neuroinflammation, oxidative stress, and degeneration of cholinergic neurons, all of which contribute to hippocampal neuron damage and memory loss [3].

Age-related diseases, including neurodegenerative and cardiometabolic disorders, have been associated with disruptions in both the cardiovascular and neurological systems, as well as alterations in behavior. Inorganic compounds, such as AlCl_3_, are believed to play a role in the pathogenesis of disorders like AD, neuroinflammation, CVD, and IR. AlCl_3_ adversely affects oxidative signaling pathways, leading to neural tissue damage, the formation of amyloid-β plaques, cognitive decline, neuroinflammation, and, ultimately, neuronal cell death. Additionally, exposure to AlCl_3_ leads to increased production of reactive oxygen species, causing lipid peroxidation, vasoconstriction, insulin resistance, and disturbances in both cardiovascular and glycemic status [4,5].

Inflammation and oxidative stress contribute to the progression and pathogenesis of neurodegenerative diseases and metabolic disturbances. Reactive oxygen species (ROS) are second messengers that modulate neuroinflammation at various stages through redox-sensitive mechanisms. Additionally, excessive ROS production or redox dysregulation under oxidative stress damages cells, leading to glucose dysregulation. A pronounced astrocyte response can produce danger signals that trigger neuroinflammation and metabolic disturbances [6].

Furthermore, exposure to AlCl_3_ increases the protein expression of IL signaling pathways and ROS, which play central roles in the hippocampus. This indicates that AlCl_3_ activates IL signaling pathways and oxidative stress. The activation of astrocytes, microglia, and ROS, along with elevated levels of pro-inflammatory cytokines, are characteristic features of AD and IR [7]. The proliferation and activation of astrocytes and microglia results in the release of pro-inflammatory cytokines, including interleukin-1 (IL-1), tumor necrosis factor-alpha (TNF-α), and interleukin-6 (IL-6). Evidence suggests that IL-6 contributes to neuroinflammation, thereby inducing neurodegenerative diseases. Additionally, overexpression of IL-6 is a common factor in the pathogenesis of neurodegenerative diseases [8].

Previous investigations have suggested that metformin holds potential to improve neuroinflammation and AD due to its ability to cross the blood–brain barrier (BBB) and its potent insulin-sensitizing action [9]. Researchers have revealed that the beneficial effects of metformin on cognitive dysfunction and memory are likely mediated through the reduction of IR and oxidative stress [10]. Likewise, a randomized controlled clinical trial demonstrated that metformin reduces the risk of microvascular and macrovascular diseases, such as myocardial infarction (MI), cardiac failure, and coronary artery disease [11,12]. Metformin’s activation of adenosine monophosphate-activated protein kinase (AMPK) represents a promising approach for managing cardiac diseases like atherosclerosis and MI. This is achieved through the improvement of mitochondrial and endothelial dysfunction, attenuation of inflammatory reactions, and reduction in oxidative stress [13,14].

IR and neuroinflammation are interconnected, leading to a pro-inflammatory state regulated by cytokines that might contribute to an increased risk of type 2 diabetes and neurodegenerative diseases. Recent research has uncovered associations between hyperglycemia, oxidative stress, inflammation, and lipoperoxidation products. These factors are linked to abnormal cellular accumulation of reactive dicarbonyl metabolites, which contribute to cell and tissue dysfunction in numerous diseases and during aging. Metformin, as evidenced by recent studies, offers protective effects against inflammation, leading to an improvement in metabolic disturbances and oxidative stress [15]. Moreover, metformin exhibits antioxidant properties and plays a neuroprotective role while also enhancing glucose metabolism. Notably, metformin has demonstrated the ability to stimulate neurogenesis and enhance spatial memory. In addition to its benefits, metformin ameliorates dyslipidemia and IR, and it reveals cardioprotective effects by reducing levels of reactive oxygen species (ROS), cytokines, interleukins (ILs), and dicarbonyl metabolites [16].

Some non-interventional studies have suggested that vitamin E may play a role in the prevention of cardiometabolic disorders, AD, and neuroinflammation. Vitamin E exhibits numerous biological actions, serving as an antioxidant to scavenge toxic free radicals. The proposition that free radicals contribute to the pathological processes underlying cognitive dysfunction has raised interest in the potential use of vitamin E for the treatment of cognitive impairment and memory deficits [17]. According to the European Society of Cardiology, CVDs remain the leading cause of mortality and morbidity in both men and women, particularly in low-income countries. Currently available information suggests that vitamin E might be critical in reducing the risk of CVDs, particularly atherosclerosis and coronary heart disease. Vitamin E is considered a vital antioxidant, primarily responsible for protecting lipids from oxidative damage [18,19]. Several studies have demonstrated the protective role of vitamin E in CVDs, attributed to its ability to regulate gene expression, cholesterol metabolism, signaling pathways, and maintaining plaque stability [18,19].

Studies have suggested that vitamin E has positive effects on cell membrane fluidity, oxidative stress, and inflammation in the brains of experimentally induced diabetic rats. Vitamin E is perceived to be effective in supporting the antioxidant and anti-inflammatory defense systems. The research revealed a reduction in the accumulation of reactive oxygen species (ROS) and a decrease in the generation of oxidative damaging substances and cytokines. Moreover, it maintained the fluidity of the brain’s membranes in the rats [20,21].

The purpose of this study was to shed light on the potential neuroprotective and cardiometabolic benefits of metformin and vitamin E in the context of AlCl_3_-induced neuroinflammation and cardiometabolic disturbance. Ultimately, the main concern of this study is to contribute to the advancement of knowledge in the field of neuroprotection and cardiometabolic health, offering promising avenues for further research and potential clinical applications.

## 2. Materials and Methods

### 2.1. Experimental Design for the Induction of Neuroinflammation and Cardiometabolic Disturbance

Approximately 30 adult Wistar rats weighing approximately 150–200 g were obtained locally and housed in the animal facility of the University of Agriculture Faisalabad (UAF), Pakistan, at an ambient temperature of 25 ± 5 °C. The rats were provided with a standard diet and had access to water ad libitum. An acclimation period of one week was allowed before commencing the experimental procedures. All experimental protocols adhered to the approved guidelines of the Institutional Biosafety and Bioethics Committee (IBC) of UAF (No. 2857/ORIC).

Following the acclimation period, the rats were equally divided into five groups (n = 6). Group 1 served as the control group and received a normal diet and water ad libitum for 45 days. Group 2 was designated as the diseased group and received AlCl_3_ at a dosage of 50 mg/kg/day. The remaining groups were also exposed to AlCl_3_ at 50 mg/kg/day and subsequently received different treatments. Group 3 received treatment I, consisting of metformin at a dosage of 100 mg/kg/day. Group 4 received treatment II, comprising vitamin E at a dosage of 150 mg/kg/day administered orally. Group 5 was the combination group, receiving both metformin at 100 mg/kg/day and vitamin E at 150 mg/kg/day.

### 2.2. Blood and Tissue Sampling

Approximately 1.5–2.5 mL of blood was collected from each rat in all groups at 0, 15, 30, and 45 days of the treatment duration using the tail vein method for biochemical analysis of relevant biomarkers. Following collection, the blood samples were centrifuged at 3000 rpm for 10–20 min to separate the serum, which was then stored at −20 °C until further analysis. At the conclusion of the study, various organ tissues, including the heart, brain, and pancreas, were dissected and separated for histopathological examination, immunochemistry, and fibrosis analysis.

### 2.3. Biochemical Analysis

The serum was utilized to measure various biochemical parameters, including the glycemic profile (glucose, insulin, and HbA1c), lipid profile (HDL, LDL, cholesterol, TG), lipid peroxidation analysis (MDA), and inflammatory biomarkers.

### 2.4. Assessment of Glycemic Status

Measuring glucose, insulin, and HbA1c levels in neuroinflammation and cardiometabolic disturbance is important because these parameters reflect the status of glucose metabolism and insulin sensitivity. Elevated glucose levels can indicate impaired glucose regulation, while insulin levels provide insights into insulin resistance and pancreatic function. HbA1c, a long-term marker of average blood glucose levels, helps assess glycemic control over time, providing valuable information about the risk of diabetes and its complications in the context of neuroinflammation and cardiometabolic disturbances.

In each group, the serum level of glucose was determined using the glucose assay kit (cat no. 1004, Elabscience) and measured with a Microlab 300 chemistry analyzer (ELITech Group) to assess the effects of metformin and vitamin E on glycemic status. Additionally, HbA1c and insulin levels in the serum were quantified using their respective ELISA kits: the insulin ELISA kit (cat no. INS 5275, Elabscience) and the HbA1c ELISA kit (cat no. SG10984, Elabscience) were utilized, and readings were obtained through a microplate ELISA reader (BIOBASE-EL 10A).

### 2.5. Assessment of Lipid Profile

Measuring the lipid profile in neuroinflammation and cardiometabolic disturbance is essential because these conditions are associated with altered lipid metabolism, which can lead to dyslipidemia. Monitoring lipid levels, including cholesterol, triglycerides, LDL, and HDL, helps assess the risk of cardiovascular diseases, which are often linked to neuroinflammation and cardiometabolic disorders. Abnormal lipid profiles can contribute to the development and progression of atherosclerosis and other vascular complications, making lipid assessment crucial for understanding the overall cardiovascular health in these conditions.

The serum levels of lipids were measured from the collected animal samples. Cholesterol (cat no. 1011, Elabscience), TG (cat no. 090618, Elabscience), HDL (cat no. 6011668, Elabscience), and LDL (cat no. 6011668, Elabscience) concentrations in the serum were quantified using a Microlab 300 chemistry analyzer.

### 2.6. Assessment of Liver Function Enzymes

The measurement of serum levels of AST (Aspartate aminotransferase) and ALT (Alanine aminotransferase) is important in neuroinflammation and cardiometabolic disturbance because these enzymes are primarily found in the liver and released into the bloodstream when there is liver damage or dysfunction. Neuroinflammation and cardiometabolic disturbances can lead to oxidative stress and tissue damage, including the liver. Elevated AST and ALT levels serve as important biomarkers to assess liver health and the extent of tissue damage in these conditions. Monitoring these liver enzymes can provide valuable insights into the overall health and potential complications associated with neuroinflammation and cardiometabolic disturbances.

The serum levels of AST, ALT, and hexokinase were measured using the AST assay kit (cat no. BD088918) and the ALT assay kit (cat no. BD088918) on the Microlab 300 chemistry analyzer. Additionally, the hexokinase levels were quantified using the hexokinase assay kit (cat No. EEL-M146, Elabscience) and read with a microplate ELISA reader.

### 2.7. Assessment of Inflammatory Biomarkers

Measuring the serum level of IL-6 in neuroinflammation and cardiometabolic disturbance is essential because IL-6 is a key pro-inflammatory cytokine that plays a crucial role in the immune response and inflammatory processes. Elevated levels of IL-6 can indicate the presence and severity of inflammation, and its measurement helps in understanding the immune and inflammatory status in these conditions. The serum level of IL-6 was measured using an IL-6 ELISA kit (E-ELR0015, Elabscience) with a microplate ELISA reader.

### 2.8. Assessment of Lipid Peroxidation Biomarker

Measuring the serum level of MDA in neuroinflammation and cardiometabolic disturbance is important because MDA is a biomarker of lipid peroxidation and oxidative stress. Elevated levels of MDA can indicate increased oxidative damage, which is known to contribute to the pathogenesis of neuroinflammation and cardiometabolic disorders. MDA, an end product of lipid peroxidation and a well-established oxidative stress biomarker, was evaluated using an MDA ELISA kit (cat no. E-EL 0060, Elabscience) with a microplate ELISA reader.

### 2.9. Analysis of ECG Pattern

Measuring the ECG pattern in neuroinflammation and cardiometabolic disturbance is crucial because it provides valuable information about the electrical activity and rhythm of the heart. Changes in the ECG pattern can indicate cardiac dysfunction or arrhythmias, which are common complications associated with neuroinflammation and cardiometabolic disorders.

After administering anesthesia, the animals from each experimental group were laid flat on the working slab. Electrodes were connected to each arm of the animal, and the standard leads were positioned on the palms of the left and right limbs. The grounded lead was attached to the right hind foot. Subsequently, the Bio Amp cable was plugged into the Bio Amp input, and electric signals were generated and recorded by a device connected to the Power-Lab.

### 2.10. Histopathological Examination of Heart, Brain, and Pancreatic Tissues

**Hematoxylin and eosin tissue staining**: Hematoxylin and eosin (H&E) tissue staining was performed in this study to examine the overall histological changes in brain, heart, and pancreatic tissues in response to neuroinflammation and cardiometabolic disturbance induced by AlCl_3_. H&E staining allowed for visualization and assessment of cellular morphology, detection of abnormalities, and evaluation of the structural integrity of the tissues, providing important insights into the impact of the treatments on tissue health. To investigate the effects of metformin, vitamin E, and the combination of metformin and vitamin E on the morphology of brain, heart, and pancreatic tissues, the rats were euthanized using cervical dislocation. Subsequently, the skull and abdomen were dissected, and the brain, heart, and pancreas were carefully separated for histopathological examination. The tissue samples were subjected to a series of standard procedures, including fixation, embedding, sectioning, mounting, and staining, following the established protocol. Briefly, slices of brain, heart, and pancreatic tissues were preserved in 10% formalin solution. The tissue sections were then washed and rehydrated using alcohol. Subsequently, all tissue sections were transferred to a xylene solution for clearing. Afterward, the sections were immersed in liquid paraffin for approximately 2 h and allowed to fix in paraffin wax. Later, the tissue sections were stained with H&E stain and covered with a glass coverslip for subsequent analysis and examination under a microscope.

**Masson’s trichrome tissue staining**: Masson’s trichrome tissue staining was performed in this study to assess the extent of fibrosis and collagen deposition in the heart and pancreatic tissues, which are associated with cardiometabolic disturbances. This staining technique allowed the researchers to visualize and quantify the fibrotic changes, providing valuable information about tissue remodeling and pathological alterations in these organs. Masson’s trichrome staining was performed to detect collagen fibers in the brain, heart, and pancreatic tissues. The tissue samples were first preserved in formalin, and paraffin-embedded sections were prepared and stored. To begin the staining process, all tissue slides were deparaffinized by heating and then rehydrated sequentially through 70%, 95%, and 100% alcohol. Distilled water was used for washing. The tissue slides were then treated with Bouin’s solution and heated at 56 °C for 1 min. Following this, they were allowed to stand for 15 min and then washed with water for 5 min. Next, the slides were stained with Weigert’s iron hematoxylin solution for 10 min and rinsed with warm water for 10 min. After washing with distilled water, the slides were immersed and stained in Biebrich scarlet acid fuchsin for 15 min. Subsequently, the tissue slides were washed with distilled water and immersed in a phosphomolybdic/phosphotungstic acid solution for 15 min. Further, the slides were stained with aniline blue solution for 10 min and rinsed with distilled water. To complete the staining process, the slides were placed in a 1% acetic acid solution for 2 min, followed by washing with distilled water. Finally, the slides were dehydrated by sequential immersion in 95% ethanol and absolute alcohol and cleared in xylene before being mounted.

**Immunohistochemistry**: Immunohistochemistry (IHC) was performed in this study to investigate the presence and distribution of specific proteins or biomarkers related to neuroinflammation and cardiometabolic disturbances in the brain, heart, and pancreatic tissues. By utilizing IHC, the researchers were able to visualize and quantify the expression of target proteins, such as amyloid plaques and neurofibrillary tangles in the brain, which are crucial in understanding the underlying mechanisms of these conditions and assessing the efficacy of the treatment interventions. Brain tissue sections, approximately 3–5 microns in thickness and embedded in paraffin, were cut and mounted onto microscopic slides. The slides were air-dried for approximately 2 h at 58 °C and subsequently underwent deparaffinization and rehydration. To facilitate heat-induced epitope retrieval, the tissue slides were exposed to an immuno-DNA retriever solution containing citrate. The slides were placed in a staining dish with the immuno-DNA retriever solution and then positioned on a trivet inside a pressure cooker. Approximately 1–2 inches of distilled water was added to the pressure cooker, and the heat was set to high. The slides were incubated under pressure for approximately 15 min. Afterward, the pressure cooker was opened, and the slides were allowed to cool to room temperature. To complete the process, the slides were mounted using biodegradable permanent mounting medium [X Green PermaMounter (BB0169-0174)].

### 2.11. Statistical Analysis

The statistical analysis of the study data was performed using ANOVA followed by post hoc tests to compare multiple groups. The results were presented as mean ± standard deviation (SD) or standard error of mean (SEM), and statistical significance was determined using *p*-values. A significance level of *p* < 0.05 was considered statistically significant. Additionally, graphs and figures were used to visualize the data distribution and trends.

## 3. Results

### 3.1. Effect of Treatment on Glycemic Status

**Figure 1:**
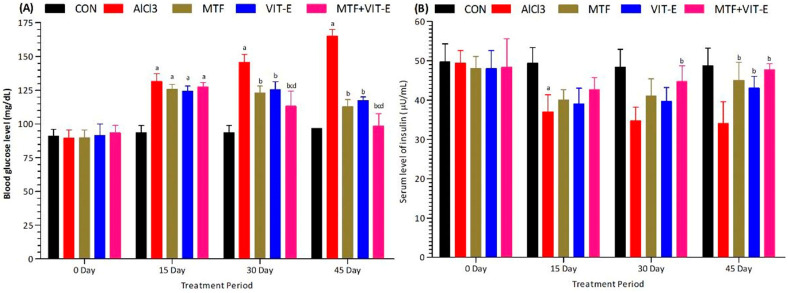
Effect of treatment on serum levels of glucose (**A**) and insulin (**B**) at 15, 30, and 45 days of the treatment period. The level of significant difference was estimated by Bonferroni post hoc test using two-way ANOVA. ^a^ represents *p* < 0.001 when compared with control group at all sampling points. ^b^ represents *p* < 0.001 when compared with AlCl_3_-treated group. ^c^ represents *p* < 0.01 when compared with MTF-treated group. ^d^ represents *p* < 0.01 when compared with VIT-E-treated group. Abbreviations|CON: Control, AlCl_3_: Aluminium chloride, MTF: Metformin, VIT-E: Vitamin E.

The effects of metformin, vitamin E alone, and their combination on serum levels of glucose (Figure 1A) and insulin (Figure 1B) were assessed at multiple time points (0, 15, 30, and 45 days) during the study duration. The experimental groups were exposed to AlCl_3_ at a dose of 50 mg/kg/day. Upon exposure to AlCl_3_, the serum levels of glucose significantly increased (*p* < 0.001) at 15, 30, and 45 days, while the insulin levels decreased significantly (*p* < 0.001) at the same time points. These findings suggest the development of insulin resistance and dysregulation of glucose homeostasis in response to AlCl_3_ exposure.

On the other hand, the groups treated with metformin and vitamin E showed interesting results. At 15 days, both metformin and vitamin E treatments led to a significant increase in serum insulin levels (*p* < 0.05) compared to the AlCl_3_-exposed group, indicating a potential improvement in insulin sensitivity. At 30 days, the combination of metformin and vitamin E resulted in a more pronounced increase in serum insulin levels compared to either treatment alone (*p* < 0.01), demonstrating a synergistic effect of the combined therapy in enhancing insulin secretion or action.

Furthermore, at 15 and 30 days, the combination group exhibited a significant decrease in serum glucose levels (*p* < 0.05) compared to the AlCl_3_-exposed group, indicating an improvement in glucose regulation. Interestingly, at 30 days of treatment duration, the combination group also showed a significant decline in serum glucose levels (*p* < 0.01) compared to both the metformin and vitamin E alone groups, indicating a superior effect of the combined treatment in lowering glucose levels. At 45 days of study duration, the metformin group showed an increased level of insulin (*p* < 0.01) and a decrease in glucose levels (*p* < 0.01) compared to the vitamin E group, suggesting that metformin has a more potent effect on insulin regulation and glucose control in the longer term.

Notably, the combination group demonstrated the most favorable outcomes, showing a significant decrease in serum glucose levels (*p* < 0.001) and a marked increase in serum insulin levels (*p* < 0.001) compared to all other groups at the 45-day study period. These results highlight the potential synergistic effects of metformin and vitamin E in improving both insulin sensitivity and glucose homeostasis, which may have implications for managing insulin resistance, cardiometabolic disorders, and Alzheimer’s disease. Nevertheless, further studies are needed to elucidate the underlying mechanisms of these effects and to assess the long-term effects of this combination therapy on overall health and disease prevention.

### 3.2. Effect of Treatment on Lipid Status

**Figure 2:**
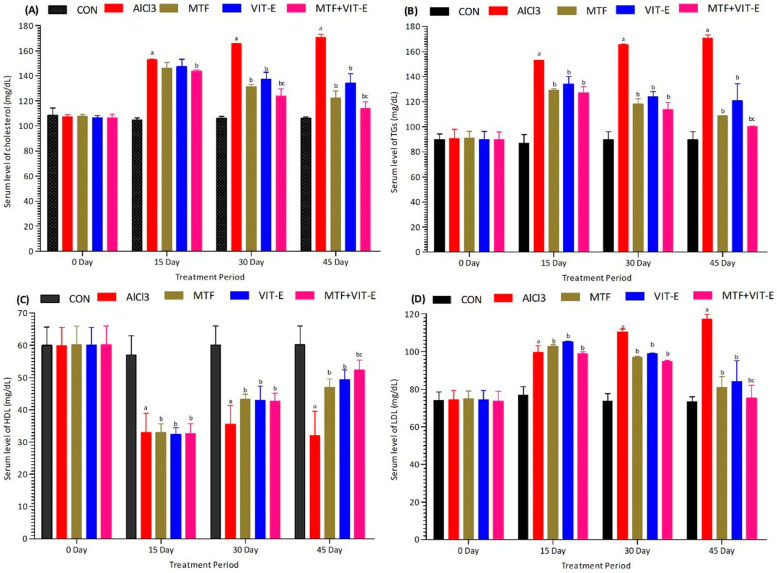
Effect of treatment on serum levels of cholesterol (**A**), TG (**B**), HDL (**C**), and LDL (**D**) at 15, 30, and 45 days of the treatment period. The level of significant difference was estimated by Bonferroni post hoc test using two-way ANOVA. ^a^ represents *p* < 0.001 when compared with control group at all sampling points. ^b^ represents *p* < 0.001 when compared with AlCl_3_-treated group. ^c^ represents *p* < 0.01 when compared VIT-E-treated group. Abbreviations|CON: Control, AlCl_3_: Aluminium chloride, MTF: Metformin, VIT-E: Vitamin E.

The effects of metformin, vitamin E alone, and their combination on serum levels of cholesterol (Figure 2A), triglycerides (TG) (Figure 2B), HDL (Figure 2C), and LDL (Figure 2D) were measured at multiple time points (0, 15, 30, and 45 days) during the study duration. The experimental groups were exposed to AlCl_3_ at a dose of 50 mg/kg/day.

After exposure to AlCl_3_, we observed a significant decrease in serum HDL levels (*p* < 0.001) and a notable increase in serum levels of cholesterol (*p* < 0.001), LDL (*p* < 0.001), and TG (*p* < 0.001). These changes in lipid profile indicate a disturbance in lipid metabolism and the development of dyslipidemia in response to AlCl_3_ exposure.

In contrast, the groups treated with metformin and vitamin E showed favorable effects on lipid levels. At 15 and 30 days, both metformin and vitamin E treatments led to a significant increase in serum HDL levels (*p* < 0.05) and a decrease in serum levels of cholesterol (*p* < 0.01), LDL (*p* < 0.01), and TG (*p* < 0.01) compared to the AlCl_3_-exposed group. These findings suggest potential benefits of metformin and vitamin E in improving lipid metabolism and reducing lipid abnormalities.

Interestingly, the combination group exhibited the most significant effects at 30 days of treatment duration. Compared to both the metformin and vitamin E alone treatment groups, the combination therapy showed maximum improvement in HDL levels (*p* < 0.001) and the most pronounced reduction in serum levels of cholesterol (*p* < 0.001), TG (*p* < 0.001), and LDL (*p* < 0.001).

At 45 days of study duration, the metformin group also demonstrated better effects (*p* < 0.01) compared to the vitamin E group in terms of improving lipid levels. However, the combination group showed the most favorable outcomes, displaying a remarkable decline in serum levels of cholesterol (*p* < 0.001), TG (*p* < 0.001), and LDL (*p* < 0.001), along with a significant elevation in serum HDL levels (*p* < 0.001) at the 45-day study period. These results suggest that the combination of metformin and vitamin E has potent lipid-regulating effects, which may be crucial in mitigating the risk of cardiometabolic disorders.

These findings highlight the potential therapeutic benefits of combining metformin and vitamin E in managing dyslipidemia and maintaining a healthy lipid profile. Nevertheless, further studies are warranted to elucidate the mechanisms underlying these effects and to assess the long-term impacts of this combination therapy on cardiovascular and metabolic health.

### 3.3. Effect of Treatment on Liver Enzymes and Glycolysis

**Figure 3:**
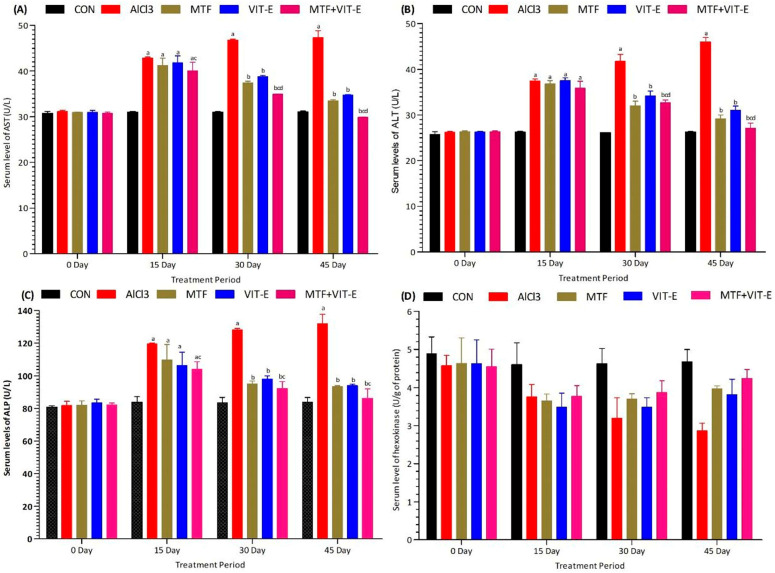
Effect of treatment on serum level of AST (**A**), ALT (**B**), ALP (**C**), and hexokinase (**D**). The effect of treatment on serum levels of AST, ALT, ALP, and hexokinase was measured at 0, 15, 30, and 45 days of the treatment period. The level of significant difference was estimated by Bonferroni post hoc test using two-way ANOVA. ^a^ represents *p* < 0.001 when compared with control group at all sampling points. ^b^ represents *p* < 0.001 when compared with AlCl_3_-treated group. ^c^ represents *p* < 0.01 when compared with MTF-treated group. ^d^ represents *p* < 0.01 when compared with VIT-E-treated group. Abbreviations|CON: Control, AlCl_3_: Aluminium chloride, MTF: Metformin, VIT-E: Vitamin E.

The effects of metformin, vitamin E alone, and their combination on serum levels of liver enzymes AST (Figure 3A), ALT (Figure 3B), ALP (Figure 3C), and hexokinase (Figure 3D) were measured at multiple time points (0, 15, 30, and 45 days) during the study duration. The experimental groups were exposed to AlCl_3_ at a dose of 50 mg/kg/day.

Following exposure to AlCl_3_, there was a significant elevation in serum levels of AST (*p* < 0.001), ALT (*p* < 0.001), and ALP (*p* < 0.001), indicating liver injury and dysfunction. Additionally, a decline in the serum level of hexokinase (*p* < 0.001) was observed, suggesting impaired glucose metabolism in the liver.

In contrast, both the metformin and vitamin E groups demonstrated beneficial effects on liver enzyme levels at 15 and 30 days. These treatments led to a significant decrease in serum levels of ALT (*p* < 0.05), AST (*p* < 0.05), and ALP (*p* < 0.05), indicating a potential improvement in liver function and reduced liver damage. Furthermore, these groups exhibited a notable increase in the serum level of hexokinase (*p* < 0.01), suggesting enhanced glucose metabolism in the liver.

Remarkably, the combination group demonstrated the most significant effects at 15 and 30 days of treatment duration. Compared to both the metformin and vitamin E alone treatment groups, the combination therapy showed more pronounced reductions in serum levels of ALT (*p* < 0.001), AST (*p* < 0.001), and ALP (*p* < 0.001), indicating a superior protective effect on liver function. Additionally, the combination group displayed the most substantial increase in serum hexokinase levels (*p* < 0.001), indicating enhanced glucose metabolism in the liver. At 45 days of study duration, the combination group continued to show the most favorable outcomes, displaying a remarkable decline in serum levels of ALT (*p* < 0.001), AST (*p* < 0.001), and ALP (*p* < 0.001), along with a significant elevation in serum hexokinase levels (*p* < 0.001). These results indicate that the combination of metformin and vitamin E has potent hepatoprotective effects, which may be crucial in mitigating liver injury induced by AlCl_3_ exposure and maintaining liver function.

These findings underscore the potential therapeutic benefits of combining metformin and vitamin E in protecting liver health and improving glucose metabolism. However, further studies are needed to elucidate the mechanisms underlying these effects and to assess the long-term impacts of this combination therapy on liver function and overall health.

### 3.4. Effect of Treatment on Inflammatory Biomarker and Lipid Peroxidation

**Figure 4:**
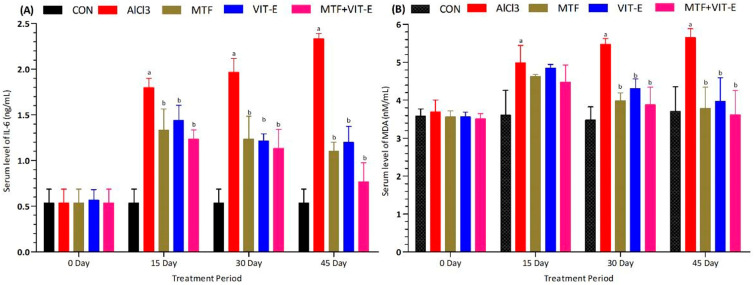
Effect of treatment on serum level of IL-6 (**A**) and MDA (**B**) at 15, 30, and 45 days of the treatment period. The level of significant difference was estimated by Bonferroni post hoc test using two-way ANOVA. ^a^ represents *p* < 0.001 when compared with control group at all sampling points. ^b^ represents *p* < 0.001 when compared with AlCl_3_-treated group. Abbreviations|CON: Control, AlCl_3_: Aluminium chloride, MTF: Metformin, VIT-E: Vitamin E.

The effects of metformin, vitamin E alone, and their combination on the inflammatory biomarker IL-6 (Figure 4A) were measured at multiple time points (0, 15, 30, and 45 days) during the study period. The experimental groups were exposed to AlCl_3_ at a dose of 50 mg/kg/day. Following the administration of AlCl_3_, there was a significant increase in the serum level of the inflammatory biomarker IL-6 (*p* < 0.001), indicating the induction of an inflammatory response. However, both the metformin and vitamin E groups showed promising results in reducing the serum level of IL-6 at 15 (*p* < 0.05) and 30 days (*p* < 0.01) of treatment duration. These findings suggest that both metformin and vitamin E have anti-inflammatory properties and can attenuate the inflammatory response induced by AlCl_3_ exposure.

Remarkably, the combination group exhibited the most significant effects at 15 and 30 days of treatment duration. Compared to both the metformin and vitamin E alone treatment groups, the combination therapy showed a more pronounced decline in the serum level of IL-6 (*p* < 0.001), indicating a synergistic anti-inflammatory effect. At 45 days of study duration, the metformin group continued to show a greater effect in decreasing the serum level of IL-6 (*p* < 0.01) compared to the vitamin E group. However, the combination group demonstrated the most potent anti-inflammatory effect, displaying a remarkable decrease in the serum level of IL-6 (*p* < 0.001) at the 45-day study period.

These results suggest that the combination of metformin and vitamin E exerts a superior anti-inflammatory effect compared to each treatment alone. The significant reduction in IL-6 levels indicates that this combination therapy may have a protective role in suppressing inflammation, which is essential in managing various inflammatory diseases and conditions. These findings hold promise for the development of novel therapeutic strategies targeting inflammation-associated conditions, such as cardiovascular diseases, neurodegenerative disorders, and other inflammatory diseases.

The effect of metformin, vitamin E alone, and their combination on the serum level of MDA was measured at multiple time points (0, 15, 30, and 45 days) during the treatment duration (Figure 4B). The experimental groups were exposed to AlCl_3_ at a dose of 50 mg/kg/day. After the administration of AlCl_3_, there was a significant increase in the serum level of MDA (*p* < 0.001), indicating the occurrence of lipid peroxidation and oxidative stress. However, both the metformin and vitamin E groups demonstrated positive effects in reducing the serum level of MDA at 15 (*p* < 0.05) and 30 days (*p* < 0.01) of the treatment period. These findings suggest that both metformin and vitamin E possess potent antioxidant properties and can effectively counteract oxidative stress induced by AlCl_3_ exposure.

Remarkably, the combination group showed the most significant effects at 15 and 30 days of treatment duration. Compared to both the metformin and vitamin E alone treatment groups, the combination therapy exhibited a more pronounced decline in the serum level of MDA (*p* < 0.001), indicating a synergistic antioxidant effect. At 45 days of study duration, the combination group continued to demonstrate the most potent effect in improving the serum level of MDA (*p* < 0.001) compared to the metformin and vitamin E groups. These results suggest that the combination of metformin and vitamin E has a sustained and robust antioxidant effect, effectively reducing oxidative stress even after prolonged treatment. These findings indicate that the combination therapy of metformin and vitamin E holds great promise in alleviating oxidative stress and lipid peroxidation, which are key factors in the pathophysiology of various diseases, including cardiovascular diseases, neurodegenerative disorders, and metabolic disorders. The antioxidant properties of metformin and vitamin E offer potential therapeutic benefits in combating lipid peroxidation and oxidative stress-related diseases and promoting overall health and well-being.

### 3.5. Histopathological Analysis

**Figure 5:**
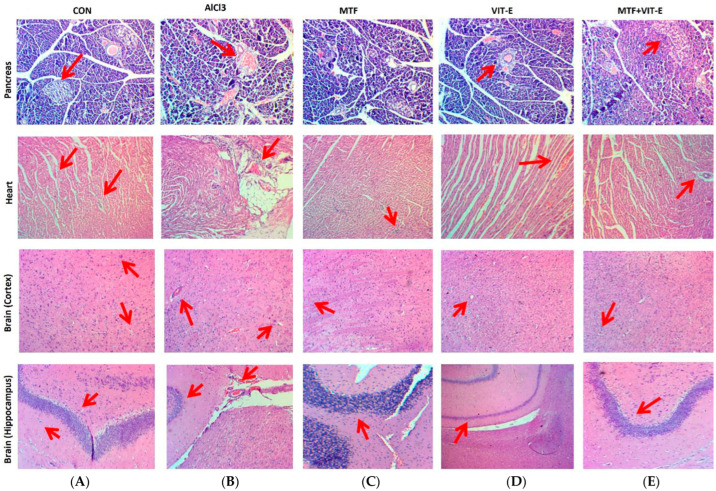
Effect of treatment after the exposure of AlCl_3_ on pancreas, heart, and brain (cortex and hippocampus) tissue morphology. In pancreas, (**A**): Islet of Langerhans appeared normal. The acinar cells seemed to be normal in structure. (**B**): Inflammation, cell swelling, and necrosis were seen. (**C**): The acinar cells appeared normal in structure. Congestion was present at a few places. (**D**): Mild shrinkage of islets of Langerhans was seen. Necrosis was present in a few sites. (**E**): Normal morphology of acinar cells and islet of Langerhans was seen. In heart, (**A**): Intercalated disk and muscle cell nucleus appeared normal. (**B**): Muscle cell nucleus disarrangement, necrosis, and inflammation were seen. (**C**): Maintenance of muscle cell nucleus was seen. (**D**): Mild necrosis was present at a few places. (**E**): Normal intercalated disk and muscle cell nucleus were seen. In brain (cortex), (**A**): Glial cells and neurons appeared normal in structure. (**B**): Granulovacuolar degeneration and ischemic neurons were seen after exposure to AlCl_3_. (**C**): Maintenance of neuronal structure was observed. (**D**): Ischemic neurons were found at a few places. (**E**): Normal neurons and glial cells were seen. In brain (hippocampus), (**A**): In control group, glial cells, neurons, and Purkinje cell cellularity showed normal appearance. (**B**): Reduced cellularity, degradation of neurons, and hemorrhage were seen after exposure to AlCl_3_. (**C**): Maintenance of Purkinje cell thickness and mild hemorrhage were seen. (**D**): Maintenance of neuronal structure but reduced cellularity of Purkinje cells were observed. (**E**): Maintenance of Purkinje cell thickness. Neurons and glial cells showed normal appearance.

The effects of metformin and vitamin E alone and in combination on pancreatic, heart, and brain tissue morphology were observed through H&E staining (Figure 5).

#### 3.5.1. Effects of Treatment on Pancreatic Tissue

The effect of metformin, vitamin E alone, and the combination of metformin + vitamin E on pancreatic tissue (Figure 5) was carefully assessed after exposure to AlCl_3_. The pancreatic tissue in the control group exhibited a normal appearance with intact islets of Langerhans, which are important for insulin production and glucose regulation. However, in the group treated with AlCl_3_, distinct morphological changes were observed in the pancreatic tissue. These changes included necrosis and shrinkage of the islets of Langerhans, indicating pancreatic toxicity and damage to the insulin-secreting cells. In contrast, the metformin- and vitamin E-treated groups showed some level of protection against pancreatic tissue damage induced by AlCl_3_. The pancreatic tissue in these groups exhibited milder necrosis compared to the AlCl_3_-exposed group, suggesting the potential protective effects of metformin and vitamin E against pancreatic toxicity.

Remarkably, the combination group displayed the most significant preservation of pancreatic tissue morphology. In this group, the islets of Langerhans were well-preserved, and signs of necrosis were notably reduced, indicating a strong protective effect of the combination therapy against AlCl_3_-induced pancreatic damage. These results suggest that the combination of metformin and vitamin E may have a synergistic effect in protecting the pancreatic tissue from the toxic effects of AlCl_3_. The observed improvements in pancreatic tissue morphology indicate the potential of this combination therapy to mitigate pancreatic toxicity and preserve the function of the islets of Langerhans in the presence of AlCl_3_ exposure.

##### 3.5.2. Effects of Treatment on Heart Tissue

The effect of metformin, vitamin E alone, and the combination of metformin + vitamin E on heart morphology (Figure 5) was carefully assessed after exposure to AlCl_3_. The heart tissue of the control group exhibited normal histology without any notable abnormalities. However, in the group treated with AlCl_3_, distinct morphological changes were observed in the heart tissue. These changes included necrosis and disarrangement of the muscle cell nucleus, indicating cardiotoxicity and damage to the cardiac tissue.

In contrast, the metformin- and vitamin E-treated groups showed some level of protection against cardiac tissue damage induced by AlCl_3_. The heart tissue in these groups exhibited milder necrosis compared to the AlCl_3_-exposed group, suggesting the potential protective effects of metformin and vitamin E against cardiotoxicity.

Remarkably, the combination group displayed the most significant preservation of heart tissue morphology. In this group, the muscle cell nuclei were well-preserved, and signs of necrosis were notably reduced, indicating a strong protective effect of the combination therapy against AlCl_3_-induced cardiac damage. These results suggest that the combination of metformin and vitamin E may have a synergistic effect in protecting the heart tissue from the toxic effects of AlCl_3_. The observed improvements in cardiac tissue morphology indicate the potential of this combination therapy to mitigate cardiotoxicity and maintain heart tissue integrity in the presence of AlCl_3_ exposure.

##### 3.5.3. Effects of Treatment on Brain Tissue

The effects of metformin, vitamin E alone, and the combination of metformin + vitamin E on brain hippocampus and cortex tissue (Figure 5) were carefully assessed. In the control group, the brain tissue displayed normal histology without any noticeable abnormalities. However, in the group treated with AlCl_3_, distinct morphological changes were observed in the brain tissue. In the hippocampus, there was reduced cellularity, degradation of neurons, and the presence of hemorrhage, indicating neurotoxicity and damage to the hippocampal region. In the cortex, granulovacuolar degeneration and signs of ischemia were observed, further confirming the detrimental effects of AlCl_3_ on cortical tissue.

In contrast, the metformin- and vitamin E-treated groups showed promising results in preserving brain tissue integrity. In the hippocampus, the brain tissue displayed maintenance of cell thickness and only mild hemorrhage, suggesting the protective effects of metformin and vitamin E against neuronal damage induced by AlCl_3_ exposure. In the cortex, mild neuronal ischemia was observed in the metformin- and vitamin E-treated groups, indicating a partial protective effect against cortical tissue damage.

Interestingly, the combination group exhibited the most notable preservation of brain tissue morphology. In the hippocampus, the combination treatment maintained the neuronal structure and thickness of Purkinje cells, demonstrating a potent protective effect against neurodegeneration and neuronal damage. In the cortex, the combination therapy showed maintenance of glial cells and reduced signs of damage, indicating a pronounced protective effect against cortical tissue injury induced by AlCl_3_. These results indicate that the combination of metformin and vitamin E has a synergistic effect in protecting brain tissue, particularly the hippocampus and cortex, against the toxic effects of AlCl_3_. The observed improvements in neuronal and glial cell integrity suggest that this combination therapy may hold promise in mitigating neurodegenerative changes and cortical damage induced by AlCl_3_ exposure.

##### 3.5.4. Masson’s Trichrome Staining after Treatment

**Figure 6:**
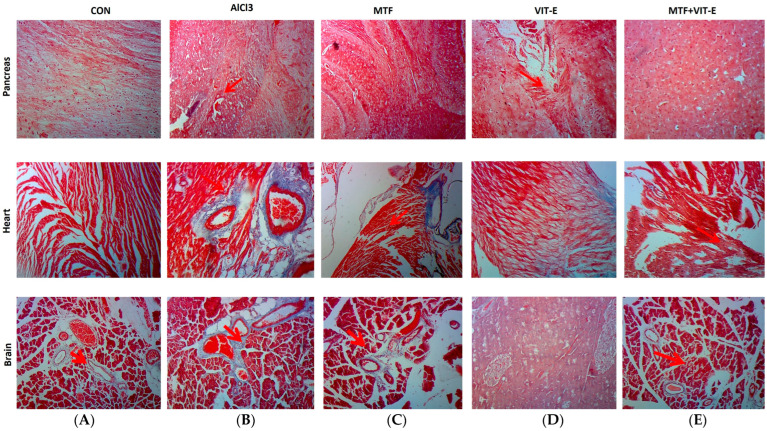
Effect of treatment on brain, heart, and pancreatic tissues after exposure to AlCl_3_ through Masson’s trichrome staining: In pancreas, (**A**): Islet of Langerhans and acinar cells seemed to be normal in structure. (**B**): Appearance of fibrotic tissues between acinar cells and around islets of Langerhans was seen after exposure to AlCl_3_. (**C**): Very mild fibrotic tissue around the islet of Langerhans was seen at a few sites of the tissue. (**D**): Few fibrotic tissues around the islet of Langerhans were present. (**E**): No fibrotic tissues around the islet of Langerhans were seen. In heart, (**A**): Cardiac muscle tissues appeared normal in structure in the control group. (**B**): Development of fibrotic tissues around the blood vessels after exposure to AlCl_3_. (**C**): Fibrotic tissues were seen. (**D**): Less collagen deposition at a few places, and no fibrotic tissues were present. (**E**): No fibrotic tissues were observed. In brain, (**A**): Normal appearance of white matter. (**B**): High vacuolation of white matter was seen after exposure to AlCl_3_. (**C**): Mild vacuolation of white matter was observed. (**D**): Vacuolation was found at a few places. (**E**): Normal neuronal structure was seen in combination group.

The effects of metformin, vitamin E alone, and in combination on brain, heart, and pancreatic tissues (Figure 6) were assessed through Masson’s trichrome staining.

###### Analysis of Fibrosis in Brain Tissue after Treatment

The control group exhibited normal brain morphology, indicating that there were no visible abnormalities in brain tissue structure in the absence of AlCl_3_ exposure. In contrast, the group treated with AlCl_3_ showed significant vacuolation of the white matter in the brain (Figure 6). Vacuolation of white matter is a characteristic feature of neurotoxicity and can lead to disruptions in neural communication and function. However, the groups treated with metformin, vitamin E alone, and the combination of both showed notable morphological improvements in brain tissue. In the metformin-treated group, we observed mild vacuolation of white matter, indicating that metformin may have a partial protective effect against AlCl_3_-induced neurotoxicity. Similarly, the vitamin E-treated group exhibited vacuolation of white matter at a few places within the tissue site, suggesting that vitamin E also offered some level of protection against neurotoxicity induced by AlCl_3_. Remarkably, the combination group displayed normal brain morphology after Masson’s trichrome staining. The brain tissue in this group showed no evidence of vacuolation, indicating that the combination therapy of metformin and vitamin E provided robust protection against AlCl_3_-induced neurotoxicity.

These findings suggest that the combination therapy may have a synergistic effect in preserving brain tissue structure and mitigating the toxic effects of AlCl_3_ on white matter. The observed improvements in brain morphology in the combination group may be attributed to the combined actions of metformin and vitamin E, which may work together to counteract the neurotoxic effects of AlCl_3_. Overall, the results from Masson’s trichrome staining indicate the potential of metformin and vitamin E combination therapy as a promising strategy to protect against neurotoxicity induced by AlCl_3_ exposure. However, further research is needed to elucidate the underlying mechanisms of this protective effect and to evaluate the functional implications of these morphological improvements in brain tissue.

###### Analysis of Fibrosis in Heart Tissue after Treatment

In this study, we investigated the effects of metformin, vitamin E, and the combination of both in a rat model after inducing neuroinflammation and cardiometabolic disturbance through the administration of AlCl_3_ (Figure 6). The control group displayed normal morphological features in heart tissue, indicating that there were no observable abnormalities in the absence of AlCl_3_ exposure.

In contrast, the group treated with AlCl_3_ (50 mg/kg/day) showed the development of fibrotic tissues around the blood vessels in heart tissue. Fibrotic tissues can impair the proper functioning of the heart and contribute to cardiovascular dysfunction. However, the groups treated with metformin and vitamin E alone exhibited mild collagen deposition and fibrotic tissue in the heart. These findings suggest that metformin and vitamin E may have a limited protective effect against AlCl_3_-induced fibrosis in the heart. Remarkably, the combination group showed maintenance of cells and no fibrotic tissue around the blood vessels in the heart. This result indicates that the combination therapy of metformin and vitamin E effectively prevented the development of fibrosis and preserved the structural integrity of heart tissue in the presence of AlCl_3_-induced neuroinflammation and cardiometabolic disturbance. The absence of fibrotic tissue in the combination group suggests a potential synergistic effect between metformin and vitamin E in protecting against cardiac fibrosis induced by AlCl_3_. The combination therapy may have targeted multiple pathways involved in fibrosis and inflammation, leading to the observed improvement in heart tissue morphology. These findings highlight the potential of the combination therapy as a promising approach to mitigating the adverse effects of AlCl_3_ on heart tissue and suggest its potential utility in preventing or ameliorating cardiometabolic disturbances associated with neuroinflammation.

###### Analysis of Fibrosis in Pancreatic Tissue after Treatment

In this study, we examined the effects of metformin, vitamin E, and their combination on pancreatic tissue after inducing neuroinflammation and cardiometabolic disturbance with AlCl_3_ (Figure 6). In the control group, the pancreatic tissue displayed a normal appearance with well-defined and intact islets of Langerhans, indicating no abnormalities in the absence of AlCl_3_ exposure. In contrast, the group treated with AlCl_3_ showed the presence of fibrotic tissues between acinar cells and around the islets of Langerhans. The development of fibrosis in the pancreas can impair the proper functioning of the organ and disrupt insulin production, leading to potential disturbances in glucose metabolism. The groups treated with metformin and vitamin E alone exhibited very mild fibrotic tissues around the islets of Langerhans. This finding suggests that both metformin and vitamin E might have a limited protective effect against AlCl_3_-induced fibrosis in the pancreas. Intriguingly, the combination group showed no fibrotic tissues around the islets of Langerhans, indicating a significant attenuation of fibrotic changes in the pancreatic tissue. This result suggests that the combination therapy of metformin and vitamin E effectively prevented the development of fibrosis and maintained the structural integrity of the islets of Langerhans in the presence of AlCl_3_-induced neuroinflammation and cardiometabolic disturbance. The absence of fibrotic tissues in the combination group indicates a potential synergistic effect between metformin and vitamin E in protecting against pancreatic fibrosis induced by AlCl_3_. The combination therapy may have targeted multiple pathways involved in fibrosis and inflammation, leading to the observed improvement in pancreatic tissue morphology. These findings underscore the potential of the combination therapy as a promising approach to mitigating the adverse effects of AlCl_3_ on pancreatic tissue and suggest its potential utility in preventing or ameliorating pancreatic fibrosis associated with neuroinflammation.

### 3.6. Analysis of ECG Pattern

**Figure 7:**
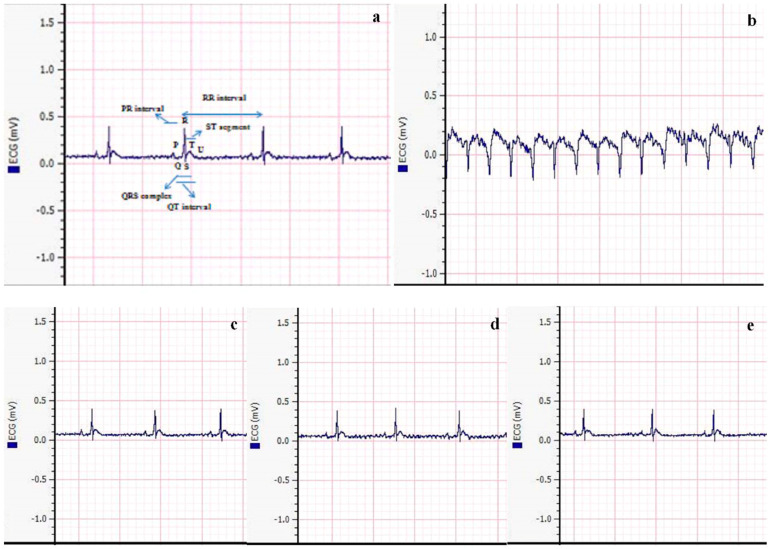
Effect of treatment on ECG after exposure to AlCl_3_: (**a**) Normal ECG pattern. (**b**) Inverted QRS complex after exposure to AlCl_3_. (**c**,**d**) Mild arrhythmia after treatment with metformin and vitamin E, respectively. (**e**) Normal ECG pattern.

In this study, we examined the effects of metformin, vitamin E, and their combination on electrocardiogram (ECG) parameters after inducing neuroinflammation and cardiometabolic disturbance with AlCl_3_ (Figure 7). In the control group, different intervals, such as QT, PR, and RR intervals, as well as the QRS complex and ST segment, exhibited a normal pattern, indicating a well-functioning cardiovascular system in the absence of AlCl_3_ exposure. However, the group treated with AlCl_3_ (50 mg/kg/day) showed an abnormality in the ECG, specifically an inverted QRS complex. The presence of an inverted QRS complex is indicative of impaired ventricular depolarization, which can lead to disturbances in cardiac function and electrical conduction.

Significant improvement in ECG findings was observed after treatment with metformin and vitamin E alone. The ECG parameters in these groups showed amelioration, indicating a positive effect on cardiac function and electrical activity. The normalization of the ECG pattern in these groups suggests that both metformin and vitamin E may have beneficial effects on the cardiac system, potentially mitigating the adverse effects induced by AlCl_3_. Interestingly, the combination group treated with metformin and vitamin E showed a completely normal ECG pattern. This finding indicates that the combination therapy effectively restored the ECG parameters to their baseline levels, resembling the ECG pattern observed in the control group. This normalization of ECG parameters in the combination group suggests a potential synergistic effect between metformin and vitamin E in protecting the cardiovascular system from the detrimental effects of AlCl_3_-induced neuroinflammation and cardiometabolic disturbance. The normal ECG pattern in the combination group further supports the idea that the combination therapy may have targeted multiple pathways involved in cardiac dysfunction and inflammation, resulting in the observed improvement in ECG findings.

These findings highlight the potential of the combination therapy as a promising approach to preserving cardiac health and preventing the adverse cardiovascular effects associated with AlCl_3_ exposure. However, further research is needed to elucidate the specific mechanisms underlying the observed cardioprotective effects and to assess the long-term safety and efficacy of the combination therapy in the context of cardiovascular diseases and related comorbidities.

### 3.7. Immunohistochemistry of Brain Tissue

**Figure 8:**
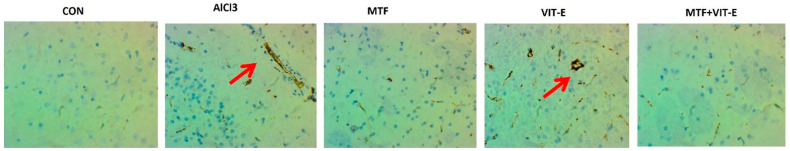
Effect of treatment after exposure to AlCl_3_ (50 mg/kg/day) on brain tissue through IHC: In control group, brain section showed normal appearance. In ALCl_3_-treated group, amyloid plaques and neurofibrillary tangles were seen after exposure to AlCl_3_. In MTF-treated group, neurofibrillary tangles were observed at a few sites after treatment with metformin. In VIT-E-treated group, amyloid plaques and neurofibrillary tangles were seen at a few places. In MTF + VIT-E-treated group, normal neuronal structure was seen.

The effects of metformin, vitamin E alone, and in combination were observed after exposure to AlCl_3_ (50 mg/kg/day) through IHC (Figure 8). The brain tissue in the control group exhibited normal histology without any evidence of abnormal protein accumulation. However, in the group treated with AlCl_3_, we observed the formation of amyloid plaques and neurofibrillary tangles in the brain tissue. These pathological features are characteristic of neurodegenerative disorders such as Alzheimer’s disease. In contrast, the metformin-treated group showed a significant improvement and decline in the number of neurofibrillary tangles. The presence of fewer neurofibrillary tangles in this group suggests a potential therapeutic effect of metformin in reducing the accumulation of these harmful protein aggregates in the brain. Similarly, the vitamin E-treated group exhibited some level of improvement, with amyloid plaques and neurofibrillary tangles observed at only a few sites in the brain tissue. This indicates that vitamin E may have a partial protective effect against the development of neurodegenerative changes induced by AlCl_3_.

Remarkably, the combination group displayed a remarkable preservation of brain tissue histology. The brain tissue in this group showed a normal histological appearance with no evidence of amyloid plaques or neurofibrillary tangles. This suggests that the combination therapy of metformin and vitamin E may have a synergistic effect in mitigating the formation of pathological protein aggregates in the brain. These results indicate the potential of the combination therapy to prevent or ameliorate neurodegenerative changes associated with aluminium exposure. However, further studies are needed to understand the underlying mechanisms of these observed effects and to explore the long-term impact of this treatment on cognitive function and neuroprotective effects. The results from the immunohistochemistry analysis demonstrate the potential of metformin and vitamin E combination therapy in preserving brain tissue histology and mitigating the development of neurodegenerative changes induced by AlCl_3_ exposure. These findings may have significant implications for the development of therapeutic interventions for neurodegenerative disorders like Alzheimer’s disease.

## 4. Discussion

In the present study, we aimed to investigate the effects of metformin, vitamin E alone, and their combination on neuroinflammation and cardiometabolic disturbance in rat models induced by daily exposure to AlCl_3_ (50 mg/kg/day, intraperitoneal) for consecutive 45 days. Aluminium is a well-known neurotoxicant that has been reported to accelerate oxidative damage, cause cell depletion in the hippocampus, and induce learning deficits. The current study focused on assessing the neuroprotective and cardioprotective effects of metformin and vitamin E in this rat model. The administration of AlCl_3_ resulted in the generation of ROS, leading to organ damage. Oxidative stress played a role in inhibiting the clearance of amyloid-β, which may contribute to its accumulation/aggregation, ultimately leading to the development of AD [22,23].

Metformin, a frequently recommended oral antidiabetic drug, has been shown to ameliorate hyperglycemia and insulin resistance without affecting low blood glucose levels. It also exhibits antioxidant and neuroprotective effects and promotes neurogenesis through the stimulation of AMP-activated protein kinase, modulating memory formation. On the other hand, vitamin E has been shown to possess anti-inflammatory, neuroprotective, and hypocholesterolemic actions [24]. A study has discovered that metformin reduces blood glucose levels through the downregulation of hepatic glucose production, a process also referred to as gluconeogenesis. It also decreases the intestinal absorption of glucose and enhances insulin sensitivity by improving peripheral glucose utilization and uptake [25]. In a recent study, it was observed that the intake of vitamin E plays a significant role in improving HbA1c and IR in a population with cardiometabolic disturbances. Vitamin E has led to decreased blood glucose levels in patients with cardiometabolic diseases [26].

Therefore, the current study aimed to evaluate the effects of metformin, vitamin E alone, and their combination on glycemic profiles. After treatment with metformin and vitamin E, there was a decrease in serum glucose levels and an increase in insulin levels at 30 and 45 days of the treatment period. The combination group showed a significant increase in insulin and a decline in glucose levels compared to other treated and control groups at 45 days of the study period (Figure 1). Metformin is known to suppress hepatic glucose production, increase peripheral glucose uptake, and enhance intestinal glucose usage [27]. Vitamin E supplementation has been shown to enhance insulin activity and decrease glucose levels due to its antioxidative effects [28].

Regarding lipid profiles, the combination group showed a significant decline in serum levels of cholesterol, triglycerides, and LDL, along with a significant increase in HDL levels at 45 days of the treatment period (Figure 2). Metformin has been reported to have positive effects on lipid profiles, reducing serum levels of cholesterol, triglycerides, and LDL while increasing HDL levels [29]. A study unveiled that metformin counters IR by preventing weight gain, reducing hyperinsulinemia, and maintaining the lipid profile. It has been discovered that metformin can also enhance the lipid profile owing to its glucose-lowering functions. Additionally, it exhibits cardiovascular protective effects [30].

Vitamin E, as a lipid-soluble chain-breaking antioxidant, exhibits anti-atherogenic and anti-inflammatory activities [31]. Observational epidemiological studies have suggested that individuals consuming high amounts of vitamin E through diet or supplements have decreased rates of cardiovascular disease [32]. Vitamin E is a potent lipid-soluble antioxidant and has been identified as a crucial component in breaking the oxidation chain reaction within membranes. It plays a significant role in safeguarding polyunsaturated fatty acids in LDL from lipid peroxidation [33].

Exposure to AlCl_3_ significantly increased the serum levels of liver function enzymes (ALT, AST, and ALP), indicating liver damage (Figure 3). The combination therapy group showed a decline in the serum levels of these enzymes and an increase in hexokinase at 45 days of the treatment period. Metformin therapy has been shown to reduce liver transaminases and improve liver function by reducing inflammation and liver fibrosis [34]. Vitamin E also plays a crucial role in maintaining liver health, as evidenced by decreased ALT levels and improved liver function in cases of liver diseases after vitamin E supplementation [31].

Inflammatory biomarkers were also assessed, and the combination group showed a more significant decline in serum levels of these biomarkers compared to metformin and vitamin E alone treatment groups (Figure 4A). Metformin has been reported to reduce the inflammatory response by restoring autophagy flux in endothelial cells and decreasing saturated fatty acid-induced lipid accumulation [35]. Elevated levels of inflammatory biomarkers in individuals with DM may increase the risk of macrovascular and microvascular complications. Anti-inflammatory therapy may prevent future complications and improve the quality of life for patients [36].

MDA is a biomarker of lipid peroxidation and oxidative stress. Increased lipid peroxidation leads to ROS formation, which can lead to neuroinflammation, myocarditis, and insulin resistance [37]. A study has discovered that a substantial number of inflammatory mediators are released during neurodegenerative diseases and metabolic disturbances. This activation of inflammatory pathways occurs in neurons, glial cells, and other bodily cells, ultimately influencing neuronal and immune excitability by modulating receptor activity and thereby contributing to neuroinflammation. Vitamin E exposure, on the other hand, reduces the risk of these diseases by functioning as an inhibitor of neuroinflammatory reactions and glial cell reactivity [38]. The combination group showed a more significant decline in serum levels of MDA at 45 days of the treatment period compared to other treated and control groups (Figure 4B). Vitamin E possesses antioxidant properties and has been reported to reduce oxidative stress, extracellular matrix deposition, and acinar cell atrophy [32].

The combination therapy of metformin and vitamin E showed significant improvements in brain and pancreatic tissue morphology compared to the effects of metformin and vitamin E alone [39]. Metformin has been shown to play a central role in inhibiting the aggregation of amyloid-β through the activation of AMPK [40]. Vitamin E has been shown to inhibit the production of ROS, improve the clearance of amyloid proteins, and prevent amyloid-β aggregation, which can ultimately help maintain neurological functions [41]. Regarding heart tissue, AlCl_3_ exposure caused myocardial damage and fibrotic tissue formation around blood vessels. However, the combination group showed no fibrotic tissue development (Figure 5). Previous studies have reported that vitamin E may be crucial in preventing cardiac fibrosis due to its anti-inflammatory and antioxidant properties. Metformin has been shown to improve endothelium-dependent microvascular response, decrease fibrotic tissue development, and ameliorate the pro-inflammatory state in atherosclerosis patients [42].

In a study by Barzegar-Amini et al. (2019), it was reported that recipients of vitamin E had a 4% lower risk of cardiac diseases compared to non-recipients [43], highlighting the protective role of vitamin E on the cardiovascular system (Figure 5). The neurotoxicity induced by AlCl_3_ exposure in rats led to cognitive dysfunction and learning and memory impairment, suggesting the development of neurological disorders. However, treatment with metformin and vitamin E showed promising effects in attenuating the formation of neurofibrillary tangles and amyloid plaques, which are characteristic features of neurodegenerative diseases. The combination group demonstrated a normal neuronal structure, indicating the potential neuroprotective effects of the combination therapy. Metformin has been shown to stimulate neurogenesis and angiogenesis while also attenuating neuroinflammation and brain atrophy, leading to functional recovery in rat models [44]. In this study, IHC was performed to determine the effect of metformin, vitamin E, and combination effects on AlCl_3_-induced neurotoxicity on brain tissue. Formation of neurofibrillary tangles and amyloid plaques was seen in the AlCl_3_ (50 mg/kg/day)-treated group. Neurofibrillary tangles and amyloid plaques were seen at a few places after treatment with metformin and vitamin E, respectively, whereas the combination group showed normal neuronal structure (Figure 8). Metformin has various properties that may be advantageous for the brain. It has been reported to stimulate neurogenesis and angiogenesis and has also been reported to attenuate neuroinflammation, decrease brain atrophy, and develop/progress functional recovery in rat models [45]. Similarly, vitamin E’s neuroprotective role has been associated with hippocampal synaptic activity, neurogenesis, and cell-signaling pathways, suggesting its potential in preventing neurodegenerative diseases by reducing oxidative stress and neuronal death [46].

Metformin’s role in activating brain-derived neurotrophic factor (BDNF) and promoting hippocampal neurogenesis has been linked to the recovery of cognitive dysfunction [47]. Dementia and Alzheimer’s disease (AD) have become significant public health concerns, with millions of individuals affected worldwide. Cognitive decline can have a measurable impact on an individual’s thinking and memory abilities, and vitamin supplementation has been suggested as a strategy to delay the development of cognitive decline in individuals with AD. Among vitamins, vitamin E has shown promise in reducing cognitive decline, acting as a scavenger of free radicals and regulating the synthesis of brain prostaglandin and nucleic acid. Some studies have predicted that vitamin E intake may slow down the progression of AD/dementia in elderly individuals [48,49]. The current study showed a significant improvement in vacuolation after continuous administration of 50 mg/kg/day AlCl_3_, with recovery observed in the combination therapy group (Figure 6). The normal neuronal structure seen in the metformin + vitamin E group indicates potential benefits in protecting against AlCl_3_-induced neurotoxicity. The heart’s network of collagen plays a crucial role in cardiac muscle contractility and provides cardiac strength. AlCl_3_ toxicities have been associated with various cardiac conditions, including myocarditis, DM, fibrosis, ischemic stroke, thrombosis, Alzheimer’s disease, pancreatitis, dementia, and necrosis [19]. Vitamin E has been identified as having anti-inflammatory and antioxidative properties, making it a potential candidate in preventing cardiac fibrosis and protecting the cardiovascular system [19]. Metformin has also shown promise in improving endothelium-dependent microvascular response, decreasing fibrotic tissue development, ameliorating the pro-inflammatory state in atherosclerosis patients, and reducing myocardial ischemia [50]. In the current study, Masson’s trichrome staining of the rat heart revealed fibrotic tissue development around blood vessels after three weeks of continuous AlCl_3_ administration, while the combination group showed no fibrotic tissue development (Figure 6). Similarly, vitamin E has been reported to prevent or alleviate the adverse effects of organic compounds on pancreatic fibrosis and collagen deposition [51]. In the present study, high fibrotic tissues were observed between acinar cells and around the islets of Langerhans after 45 days of continuous AlCl_3_ administration. However, no fibrotic tissues were observed in the combination therapy group (Figure 6), indicating the potential protective effect of metformin + vitamin E on pancreatic tissue. Furthermore, long-term exposure to AlCl_3_ has been associated with cardiac rhythm disturbances, including hypotension, chest discomfort, CNS depression, tachycardia/bradycardia, ventricular and atrial dysrhythmias, shortness of breath, coma, and even death [51,52]. Combination therapy was effective in attenuating AlCl_3_-induced alterations in the ECG pattern compared to metformin and vitamin E alone (Figure 7). These findings suggest the potential of the combination therapy to protect against AlCl_3_-induced cardiac rhythm disturbances. Oxidative stress is involved in the pathogenesis of CVDs, such as heart failure, hypertension, and atherosclerosis. The production of ROS is associated with cardiac arrhythmias. ROS and oxidative stress play roles in the development of ventricular fibrillation (VF) and ventricular tachycardia (VT). On the other hand, vitamin E has shown a significant antioxidant role in the treatment of arrhythmias related to QT interval. It has the potential to improve therapeutic outcomes in patients with ischemic heart disease and/or myocardial ischemia–reperfusion syndrome [53]. Research has reported that metformin has cardioprotective effects, including reducing myocardial infarct size, exerting positive effects on cardiac remodeling, preserving myocardium, and enhancing left ventricular functional activity [54,55].

## 5. Conclusions

The objective of this study was to illuminate the potential neuroprotective and cardiometabolic advantages of metformin and vitamin E in the context of AlCl_3_-induced neuroinflammation and cardiometabolic disturbances. Through the assessment of diverse physiological parameters, lipid profiles, inflammatory markers, and tissue morphology, this research aimed to offer valuable insights into the protective effects of these interventions against the deleterious effects of AlCl_3_ on the brain, heart, and pancreas. The administration of AlCl_3_ resulted in significant alterations in glycemic profiles, lipid profiles, liver function enzymes, inflammatory biomarkers, and oxidative stress, indicating the development of cardiometabolic disturbance and neuroinflammation. However, treatment with metformin and vitamin E alone and in combination showed promising effects in mitigating these adverse effects.

These findings suggest that combination therapy may offer enhanced glycemic control in cardiometabolic disorders. In terms of lipid profiles, the combination therapy showed the most significant improvement, with declines in serum levels of cholesterol, triglycerides, and LDL and an increase in HDL levels at 45 days of treatment. The evaluation of brain tissue morphology revealed that the combination therapy preserved neuronal structure. Similarly, the combination therapy showed positive effects on cardiac tissue morphology, with no fibrotic tissues observed around blood vessels. In summary, this study provides compelling evidence supporting the neuro- and cardioprotective effects of metformin and vitamin E alone, with the combination therapy showing the most significant overall improvements. The combination of metformin and vitamin E holds promise as a potential therapeutic strategy for mitigating cardiometabolic disturbance and neuroinflammation-induced damage, offering a novel approach to the prevention and management of neurodegenerative and cardiovascular diseases. Further research is warranted to elucidate the precise mechanisms underlying these effects and to explore the potential clinical applicability of this combination therapy in humans.

## Data Availability

All data are available within the manuscript.
